# Vitiligo: a complex disease and a complex approach

**DOI:** 10.1186/1755-8166-7-S1-I57

**Published:** 2014-01-21

**Authors:** Rasheedunnisa Begum, Yogesh S Marfatia, Naresh C Laddha, Mitesh Dwivedi, Mohmmad Shoab Mansuri, Mala Singh

**Affiliations:** 1 Department of Biochemistry, Faculty of Science, The Maharaja Sayajirao University of Baroda, Vadodara, India; 2 Department of Skin & V.D., Faculty of Medicine, The Maharaja Sayajirao University of Baroda, Vadodara, India

## 

Vitiligo is an acquired, circumscribed hypomelanotic skin disorder, characterized by milky white patches due to loss of functional melanocytes from the epidermis. Prevalence of vitiligo is found to be very high in Gujarat i.e., ~8.8%. Vitiligo is a multifactorial polygenic disorder with a complex pathogenesis, linked with both genetic and non-genetic factors. Several theories have been proposed to explain the etiopathogenesis of vitiligo, but none of the hypotheses explains the entire spectrum of this disorder. We are addressing this complex disease in our Gujarat population with various approaches. Our study mainly deals with the evaluation of oxidative stress, autoimmune, genetic and neurochemical hypotheses in Gujarat vitiligo patients. We have shown that our vitiligo patients exhibit significant oxidative stress and thus, systemic oxidative stress could play a pathophysiological role in precipitation of vitiligo in Gujarat population. Our studies revealed that presence of increased antimelanocyte antibodies and the imbalance of T-cell (CD4^+^/CD8^+^ and Tregs) subsets along with their functional defects might result in melanocyte destruction in vitiligo patients. Our results on selected candidate genes in conferring oxidative stress and autoimmunity suggest that HLA-A*33:01, HLA-A*02:01,HLA-B*44:03, HLA-DRB1*07:01 and a few studied polymorphisms in *IL4*, *CTLA4*, *SOD2*, *SOD3*, *GPX1*, *NALP1*, *MYG1*, *TNFA*, *TNFB*, *IFNG* and *IL10* genes are strongly associated with vitiligo susceptibility, whereas a few studied polymorphisms in *PTPN*22, *MBL*2, *ACE*, *CAT*, *G6PD* and *SOD1* genes are not found to be significantly associated with Gujarat vitiligo patients. Gene expression studies of the *IL4*, *CTLA4*, *SOD2*, *SOD3*, *NALP1*, *MYG1*, *TNFA*, *TNFB*, *IFNG*, *IL10* and *ICAM1* genes suggest that these genes are strongly associated with vitiligo susceptibility. We are also addressing the role of immune-regulatory genes with respect to their expression in skin along with the effect of selected cytokines on *in vitro* cultured melanocytes derived from healthy and vitiliginous human skin to have an insight towards vitiligo pathogenesis. We are also exploring the potential microRNAs involved in pathogenesis of vitiligo. This integrated study will provide a better understanding of the role played by oxidative stress and autoimmunity in the pathogenesis of vitiligo in Gujarat population and also to develop selective therapy and the genetic marker/s for vitiligo.

Various factors such as the antioxidant status, LPO (oxidative stress) levels and antimelanocyte antibody titer decide the selective therapy for our vitiligo patients. The pathogenesis of vitiligo though partially understood still remains complex and enigmatic to a greater extent. Though the condition may be precipitated by multiple etiologies, the interaction of oxidative stress and immune system clearly appears to be the key convergent pathway that initiates and/or amplifies the enigmatic loss of melanocytes in vitiligo.

